# Epidemiology of Respiratory Pathogens among Elderly Nursing Home Residents with Acute Respiratory Infections in Corsica, France, 2013–2017

**DOI:** 10.1155/2017/1423718

**Published:** 2017-12-17

**Authors:** Shirley Masse, Lisandru Capai, Alessandra Falchi

**Affiliations:** EA7310, Laboratoire de Virologie, Université de Corse-Inserm, 20250 Corte, France

## Abstract

**Background:**

The current study aims to describe the demographical and clinical characteristics of elderly nursing home (NH) residents with acute respiratory infections (ARIs) during four winter seasons (2013/2014–2016/2017), as well as the microbiological etiology of these infections.

**Methods:**

Seventeen NHs with at least one ARI resident in Corsica, France, were included. An ARI resident was defined as a resident developing a sudden onset of any constitutional symptoms in addition to any respiratory signs. Nasopharyngeal swabs from ARI residents were screened for the presence of 21 respiratory agents, including seasonal influenza viruses.

**Results:**

Of the 107 ARI residents enrolled from NHs, 61 (57%) were positive for at least one of the 21 respiratory pathogens. Forty-one (38.3%) of the 107 ARI residents had influenza: 38 (92%) were positive for influenza A (100% A(H3N2)) and three (8%) for influenza B/Victoria. Axillary fever (≥38°C) was significantly more common among patients infected with influenza A(H3N2).

**Conclusion:**

The circulation of seasonal respiratory viruses other than influenza A(H3N2) seems to be sporadic among elderly NH residents. Investigating the circulation of respiratory viruses in nonwinter seasons seems to be important in order to understand better the dynamic of their year-round circulation in NHs.

## 1. Introduction

Acute respiratory infections (ARIs) are the most common infections in humans of all ages, but the elderly are at increased risk of morbidity and mortality because of coexisting chronic disease and immunosenescence [1].

The aging of the population, especially in high income countries, has modified the landscape of respiratory infections, but few studies have described the epidemiology of ARIs in the elderly [2–9]. Each year, influenza and respiratory syncytial virus (RSV) are responsible for the deaths of more than 50,000 elderly individuals in the United States [10]. ARIs caused by other respiratory agents, including human rhinovirus (HRV), human metapneumovirus (HMPV), human coronavirus (HCoV), human parainfluenza virus (HPIV), and human adenovirus (HAdV), have also been described, but the data are scarce and fragmentary [2–9]. This lack of data could be because of the atypical presentation of ARI in elderly patients, which complicates and potentially delays clinical and laboratory diagnoses, as well as low viral loads and difficulties in implementing laboratory-based surveillance systems in nursing homes (NHs) [11, 12].

Facilities that serve vulnerable populations need special public health attention; therefore, we conducted a surveillance study of ARIs in Corsican NHs [6]. The current study aims to describe the demographical and clinical characteristics of patients with ARIs in NHs during four winter seasons (2013/2014–2016/2017), as well as the microbiological etiology of these infections.

## 2. Materials and Methods

### 2.1. Study Site and Patient Enrollment

As previously reported [6], NHs in Corsica were invited to participate in an ongoing daily epidemiological and microbiological surveillance study for ARIs among residents during four consecutive winter seasons (2013/2014–2016/2017). Participation was voluntary and unrestricted.

Each season, enrollment takes place from October to April. A resident was defined as a person with a registered home address in a NH. A case of ARI was defined as a person developing sudden onset of any constitutional symptom, in addition to any respiratory sign. Fever was considered to be present when axillary temperature was ≥38°C. Nasopharyngeal samples were collected from all participants who developed an ARI during the study. Nasopharyngeal flocked swabs were collected. ARI detection was carried out by nurses, and the diagnoses were confirmed by a physician. Patient information, including demographic characteristics (sex, age), symptoms, risk factors of severe influenza, treatment, influenza vaccination status, and hospitalization, was documented in case report forms (CRFs). The risk factors associated with severe influenza infection were chronic disease and obesity (Body Mass Index > 40 kg/m^2^). The nasopharyngeal swabs and CRFs were sent by mail daily to the virology laboratory of the University of Corsica [6]. Seventeen NHs enrolled at least one ARI resident, with a total catchment population of 1113 and a mean of 65.5 (min = 56.7 and max = 75.1). Laboratory results were communicated to physicians 24 hours after the reception of nasopharyngeal samples in the laboratory.

### 2.2. Laboratory Method

#### 2.2.1. Nucleic Acid Extraction

Nucleic acids were extracted from samples stored in 200 *μ*l of universal transport medium and eluted in 60 *μ*l of elution buffer using QiaAmp MinElute virus spin kits (Qiagen, France) according to the manufacturer's instructions. An internal control (T4 and MS2 phages) was added to each extraction tube to assess the quality of the extraction at the end of the amplification [13].

#### 2.2.2. Detection of Influenza Viruses

All samples were tested for influenza viruses A ((A(H3N2) and A(H1N1)pdm09)) and determination of influenza B virus lineage [14, 15] using real-time Reverse Transcription quantitative PCR (RT-qPCR).

#### 2.2.3. Detection of Other Respiratory Pathogens

The presence of the following noninfluenza respiratory pathogen groups was analyzed by RT-qPCR using the Fast Track Diagnostics Respiratory Pathogens 21 Kit (Fast Track Diagnostic, Luxembourg): HRV, human coronaviruses NL63 (HCoV-NL63), 229E (HCoV 229E), OC43 (HCoV-OC43), HKU1 (HCoV HKU1), RSV A/B, HMPV A/B, HAdV, HPIV-1, HPIV-2, HPIV-3, and HPIV-4, human bocavirus (HBoV), enterovirus (EV), human parechovirus (HPeV), and* Mycoplasma pneumoniae*.

### 2.3. Statistical Analyses

Categorical variables were summarized with frequencies and percentages, and numerical variables were summarized with medians and interquartile ranges. The independent *t*-test was used to compare continuous data, and the chi-square test was used to compare categorical data. Attack rates for each outbreak were calculated by dividing the total number of ARI cases among residents by the total number of residents in the NH during the outbreak. All data were analyzed using Epi Info v7 [16].

### 2.4. Ethics

All data were coded and tested anonymously. None of the authors collected samples. Patient information was stored according to national regulations (ethics committee ref 14-078), and access to such data was restricted. Informed consent was obtained from all participants. The study protocol conformed to the ethical guidelines of the 1975 Declaration of Helsinki.

## 3. Results

### 3.1. Characteristics of Residents with ARIs and Respiratory Pathogens Distribution

From December 1, 2013, to April 16, 2017, 107 residents with ARI were enrolled from 17 sentinel NHs. The proportion of residents diagnosed with an ARI and sampled was 9.6% (107/1113). The basic demographical and clinical data are shown in Table 1. Of these residents, 80 (74.8%) were women, and their median age was 88 years (interquartile range = 63–103). Fifty-five (51.4%) residents had at least one risk factor of developing severe influenza. Overall, 97 (90.6%) residents had been vaccinated against seasonal influenza. Fever (82; 76.6%), cough (96; 89.7%), and asthenia (58; 54.2%) were the most common symptoms. Physician prescribed antibiotics to 39 residents (36.4%). Antiviral (oseltamivir) treatment was prescribed to four residents (3.7%). Six residents had been hospitalized (5.6%), and three (2.8%) died (Table 1).

### 3.2. Prevalence of Respiratory Pathogens

**Figure 1:**
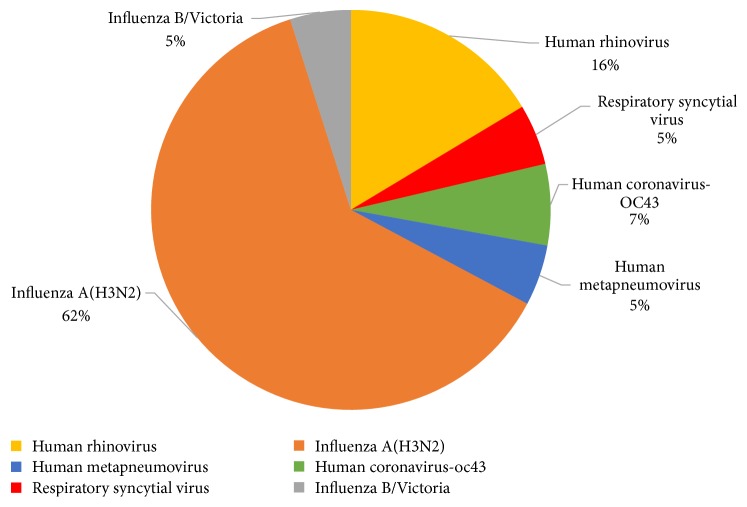
The percentages of identified viral pathogens among residents with acute respiratory infections testing positive (*N* = 61) for at least one of the 21 respiratory agents investigated in the present study.

Of the 107 ARI residents enrolled, 61 (57%) were positive for at least one of the 21 target respiratory pathogens screened (Table 2 and Figure 1). Among these residents, none had coinfection. Forty-one (38.3%) had influenza: 38 (92%) were positive for influenza A (100% A(H3N2)) and three (8%) for influenza B/Victoria. Influenza A(H3N2) circulated in NHs in all four winter seasons. Respiratory agents other than influenza viruses were detected in 20 ARI residents (18.6%). Rhinovirus was detected most frequently (10/107; 9.3%), followed by HCoV (4/107; 3.7%; 100% OC43), RSV (3/107; 2.8%), and HMPV (3/107; 2.8%).

### 3.3. Characteristics of ARI Residents with Influenza A(H3N2)

Females (median age = 88.4 years; range, 63–103 years) accounted for 73.7% of the 38 patients with influenza A(H3N2) infection. The clinical features of the participants were further examined by comparing those with confirmed influenza A(H3N2) with those with noninfluenza A(H3N2), those with respiratory viruses other than influenza (A and B), and those without any identified etiology (Table 3). Fever symptom was significantly more common among ARI residents infected with influenza A(H3N2) than among those with noninfluenza A(H3N2) (*p* = 0.004), those with respiratory viruses other than influenza (*p* = 0.002), and those without any identified etiology (*p* = 0.02) (Table 3). A significant difference in the death rate was observed between ARI residents who tested positive for influenza A(H3N2) and those with noninfluenza A(H3N2) (*p* = 0.04) (Table 3).

### 3.4. Circulation of Respiratory Pathogens

**Figure 2:**
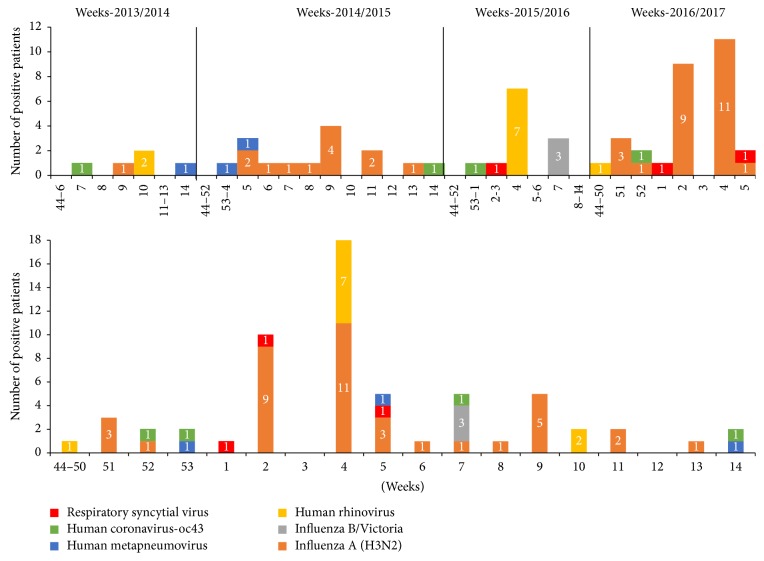
Follow-up of respiratory viruses detected by seasons and per week and cumulative total of viruses detected over the four winter seasons of surveillance (2013/2014–2016/2017).

As shown by the cumulative number of positive ARI cases by virus and week over the 4-year surveillance period (Figure 2), the highest number of positive cases (29.5%; 18/61) was identified in January (week 4). Of the 18 positive cases, 11 were influenza A(H3N2) (detected during the 2016/2017 influenza outbreak), and seven were HRV (detected during the 2015/2016 influenza outbreak). Of the 11 cases with influenza A(H3N2), four were sampled from four residents living in the same NH during the 2016/2017 influenza outbreak. The outbreak, which was limited to residents, lasted for 7 days, with an attack rate of 13.7% (5/37) among exposed residents. The seven cases of HRV belonged to the same outbreak and lasted for 4 days, with an attack rate of 7% (5/70). The outbreak was limited to residents.

## 4. Discussion

In this study, we described the clinical and microbiological characteristics of ARI residents in NHs across winter seasons for a four-year period. Influenza A(H3N2) was the most common viral pathogen detected among ARI residents, followed by HRV. The circulation of other seasonal influenza viruses among ARI residents was sporadic. Fever symptoms seemed to be predictor of a confirmed influenza A(H3N2) infection.

Respiratory pathogens were detected in 57% of the ARI residents using the multiplexed real-time RT-PCR method. This detection rate of 57% was similar to what has been reported in elderly persons in a number of previous studies (40–57.6%) using a similar RT-qPCR method [2, 3, 7, 17]. In line with a recent study [7], no coinfections were detected among ARI residents. Although the detection of respiratory viruses using RT-qPCR is a highly sensitive method, there is a potential bias for detection, as viral loads in samples from the elderly are generally lower than those in samples from younger adults [18].

In this study, the most frequently detected respiratory virus was influenza A(H3N2), even though the seasonal influenza vaccination rate was 90% among the ARI residents. In France and other European countries, the 2014/2015 and 2016/2017 influenza outbreaks were characterized by the genetic evolution of circulating A(H3N2) strains and by reports of low-to-moderate influenza vaccine effectiveness in the elderly [19, 20]. Moreover, as previously described [6], the suboptimal influenza vaccination coverage of healthcare providers and the suboptimal antiviral strategies applied (less than 5%) could increase the vulnerability of NH residents to influenza infection. In France, when an influenza outbreak is suspected in an institutionalized setting, antiviral drug treatment is recommended for those individuals exposed [21]. In the present study, even when the virological results had been communicated to a physician within 24 hours after reception of the nasopharyngeal sample, influenza antiviral treatment was only administrated to three patients. These results are in agreement with the low prescription rate of neuraminidase inhibitors to patients with a severe influenza risk factor reported in French primary healthcare settings [22].

To improve vaccine efficacy in the elderly, the use of recombinant, high-dose, or adjuvanted influenza vaccination has recently been investigated [23]. A high-dose inactivated split-virus influenza vaccine was found to be more efficacious than was a standard dose for preventing laboratory-confirmed influenza illness in adults ≥65 years of age [23]. Although this high-dose vaccine is recommended in the United States, French health authorities have not issued any recommendation regarding its use.

In the present study, the circulation of other seasonal influenza viruses was sporadic among ARI residents. Influenza B/Victoria viruses, which dominated during the 2015/2016 influenza outbreak [24], were not included in the trivalent influenza seasonal vaccine and, in this study, were only detected in three residents. This is in line with knowledge that influenza B, when present as a seasonal circulating virus within a geographic area, mainly occurs among younger persons and school-aged children [25]. Although the A(H1N1)pdm09 viruses circulated in France during the 2013-2014 (47%), 2014-2015 (19%), and 2015-2016 (19%) influenza outbreaks, no A(H1N1)pdm09 positive samples were detected among the ARI residents enrolled in the present study. This result is in agreement with previous studies reporting the apparent limited circulation of A(H1N1)pdm09 in NHs [2, 26, 27] as a consequence of a degree of cross-protection acquired by residents from previous exposure to influenza A(H1N1) virus, particularly with strains that circulated before 1957 [28].

In this study, the circulation of very serious respiratory pathogens in the elderly, such as HRV and RSV [2, 4, 5], was reported among 9.8% and 2.8% of ARI residents, respectively. RSV was associated with sporadic circulation throughout the surveillance period, while HRV, the most commonly detected virus second to influenza, was associated with a mild outbreak in NHs during the 2015/2016 influenza outbreak. We found that the presence of fever remained important in the elderly to retain specificity for the diagnosis of influenza A(H3N2). This result is in agreement with those from previous studies [11, 29–31], in which fever was associated with influenza among older adults. The number of deaths was significantly associated with influenza A(H3N2)-positive ARIs residents. This result was not surprising, as the circulation of influenza virus, in particular subtype A(H3N2), has been shown to be the main seasonal driver of excess mortality, particularly among the elderly (≥65 years of age) [32, 33].

This study has some limitations. The small sample size of ARI residents reduced the statistical power of any comparison by subgroups, especially for respiratory viruses other than influenza A(H3N2). Syndromic diagnosis of ARI is often complex in elderly patients as a consequence of preexisting diseases, complications, and atypical manifestation of ARIs, and it thus remains a challenge for physicians [34]. These difficulties could lead to biased clinical assessments and decisions regarding whether to take a swab, thus underestimating the number of positive patients. Although RT-qPCR is a valid diagnostic assay with high sensitivity and specificity for respiratory viruses, the clinical implications of positive laboratory results are less straightforward. The lack of data on the etiologies of ARIs in nonwinter seasons was also a limitation of this study.

Nevertheless, two potential clinical applications can be highlighted. First, the prescription of antiviral therapy to influenza patients was extremely low, and mortality was highest in those with confirmed influenza infection. Therefore, the results may facilitate earlier antiviral therapy in influenza patients, thereby reducing mortality. Second, once positive laboratory diagnoses are made, infection control measures can be implemented with improved compliance, potentially reducing outbreaks in NHs.

## 5. Conclusions

The circulation of seasonal respiratory viruses other than influenza A(H3N2) seems to be sporadic among NH residents. Investigating the circulation of respiratory viruses in nonwinter season is therefore important to understand better the dynamic of their year-round circulation in NHs.

## Figures and Tables

**Table 1:** Characteristics of residents with acute respiratory infections (ARIs) swabbed during the four winter seasons of surveillance (2013/2014–2016/2017).

Characteristics	*N* (%)
*Number of residents swabbed*	107
*2013/2014*	6 (5.6)
*2014/2015*	29 (27.1)
*2015/2016*	26 (24.3)
*2016/2017*	46 (43.0)
*Age (years), median (IQR* ^*∗*^)	88 (63–103)
*Age group (years)*	
60–69	4 (3.7)
70–79	10 (9.3)
80–89	47 (44)
≥90	46 (43)
*Female gender, n (%)*	80 (74.8)
*Seasonal influenza vaccination, n (%)*	97 (90.6)
*Risk factors, n (%)*	55 (51.4)
*Symptoms, n (%)*	
Fever	82 (76.6)
Cough	96 (89.7)
Headache	21 (19.6)
Short of breath	15 (14.0)
Runny nose	38 (35.5)
Asthenia	58 (54.2)
Otitis	0 (0.0)
Conjunctival hyperemia	4 (3.7)
Abdominal pain	2 (1.9)
Diarrhea	3 (2.8)
Vomiting	0 (0.0)
*Antibiotics, n (%)*	39 (36.4)
*Oseltamivir, n (%)*	4 (3.7)
*Hospitalization, n (%)*	6 (5.6)
*Death, n (%)*	3 (2.8)

^*∗*^IQR: interquartile range.

**Table 2:** The number and percentage of respiratory pathogens detected in the 107 elderly nursing home residents with acute respiratory infections swabbed during the four winter seasons of surveillance (2013/2014–2016/2017).

Respiratory pathogens	Total *N* = 107	%
Positive for any virus	61	57
*Influenza A and B*	41	38.3
* Influenza A(H3N2)*	38	35.5
* Influenza B/Victoria*	3	2.8
*Respiratory viruses other than influenza*	20	18.6
* Human rhinovirus*	10	9.3
* Human coronavirus*	4	3.7
* Human metapneumovirus*	3	2.8
* Respiratory syncytial virus*	3	2.8
* Human bocavirus*	0	0
* Human adenovirus*	0	0
* Human parechovirus*	0	0
* Enterovirus*	0	0
* Human parainfluenza*	0	0
* Mycoplasma pneumoniae*	0	0
*Coinfections*	0	0

**Table 3:** Comparison of the characteristics between residents with acute respiratory infections (ARIs) testing positive and negative for influenza A(H3N2) and positive to respiratory viruses other than influenza and negative for any other identified etiology.

Characteristics	Influenza A(H3N2) cases *N* = 38 (%)	Noninfluenza A(H3N2) cases *N* = 69 (%)	*p* value	Respiratory viruses other than influenza (A and B) *N* = 20 (%)	*p* value^*∗*^	Negative (any identified etiology) cases *N* = 46 (%)	*p* value^*∗∗*^
*Age (years), median (IQR)*	87.5 (69–96)	88.4 (63–103)	0.81	83 (63–95)	0.6	90 (66–103)	0.3
*Age groups (years)*							
*60–69*	1 (2.6)	3 (4.3)	1	2 (10)	0.2	1 (2.2)	0.7
*70–79*	6 (15.7)	4 (5.7)	0.16	2 (10)	0.7	2 (4.3)	0.08
*80–89*	16 (42.1)	31 (44.9)	0.8	11 (55)	0.4	19 (41.3)	0.56
*≥90*	15 (39.4)	31 (44.9)	0.8	5 (25)	0.3	24 (52.2)	0.17
*Gender*							
Female	28 (73.7)	52 (75.3)	0.4	16 (80)	0.8	33 (71.7)	0.41
*Seasonal influenza vaccination*	36 (94.7)	61 (88.4)	0.1	18 (90)	0.6	40 (86.9)	0.1
*Risk factors*	19 (50.0)	36 (52.1)	0.4	13 (65)	0.4	23 (50.0)	0.6
*Symptoms*							
Fever	35 (92.1)	47 (68.1)	**0.004**	12 (60)	**0.002**	33 (71.7)	**0.02**
Cough	32 (84.2)	64 (92.7)	0.3	19 (95)	0.4	42 (91.3)	0.2
Headaches	11 (28.9)	15 (21.7)	0.4	4 (20)	0.5	11 (23.9)	0.4
Dyspnea	4 (10.5)	11 (15.9)	0.5	3 (15)	0.6	6 (13.0)	0.5
Rhinorrhea	15 (39.5)	23 (33.3)	0.5	9 (45)	0.7	14 (30.4)	0.3
Asthenia	18 (47.4)	40 (57.9)	0.3	12 (60)	0.4	25 (54.3)	0.3
Otitis	0 (0.0)	0 (0.0)	1	0 (0.0)	1	0 (0.0)	1
Conjunctival hyperemia	3 (7.9)	1 (1.4)	0.12	0 (0.0)	0.5	1 (2.2)	0.2
Abdominal pain	1 (2.6)	1 (1.4)	1	0 (0.0)	1	1 (2.2)	0.7
Diarrhea	0 (0.0)	3 (4.3)	0.5	1 (5)	1	2 (4.3)	0.3
Vomiting	0 (0.0)	0 (0.0)	1	0 (0.0)	1	0 (0.0)	1
*Hospitalization*	4 (10.5)	2 (2.8)	0.18	2 (10)	1	0 (0.0)	0.04
*Oseltamivir*	3 (7.9)	1 (1.4)	0.12	1 (5)	1	0 (0.0)	0.09
*Antibiotics*	13 (34.2)	26 (37.7)	0.8	12 (60)	0.09	12 (26)	0.3
*All-cause mortality*	3 (7.9)	0 (0.0)	**0.04**	0 (0.0)	0.5	0 (0.0)	0.09

*Note*. Comparison of influenza A(H3N2) cases ^*∗*^versus other respiratory viruses (influenza B viruses were not included) and ^*∗∗*^versus cases testing negative for any identified etiology. IQR: interquartile range.
